# Nutritional Value, Volatile Components, Functional Metabolites, and Antibacterial and Cytotoxic Activities of Different Parts of *Millettia speciosa* Champ., a Medicinal and Edible Plant with Potential for Development

**DOI:** 10.3390/plants12223900

**Published:** 2023-11-19

**Authors:** Wei Wang, Yigang Yan, Yitong Li, Yinyin Huang, Yirong Zhang, Lan Yang, Xiaoli Xu, Fengqi Wu, Bing Du, Ziling Mao, Tijiang Shan

**Affiliations:** 1 College of Forestry and Landscape Architecture, South China Agricultural University, Guangzhou 510642, China; 18819465384@163.com (W.W.); yyg17667412101@163.com (Y.Y.); yitong0705@163.com (Y.L.); fengqiwu@scau.edu.cn (F.W.); 2 College of Plant Protection, South China Agricultural University, Guangzhou 510642, China; zhang18838064508@163.com; 3 Affiliated Stomatology Hospital, Guangzhou Medical University, Guangzhou 510182, China; 2023686011@gzhmu.edu.cn (Y.H.); yangl1900@163.com (L.Y.); 4 Instrumental Analysis and Research Center of SCAU, South China Agricultural University, Guangzhou 510642, China; xuxiaoli@scau.edu.cn; 5 College of Food Science, South China Agricultural University, Guangzhou 510642, China; dubing@scau.edu.cn

**Keywords:** nutritional value, volatile components, functional metabolites, antimicrobial activity, cytotoxic activities

## Abstract

Highly nutritious traditional plants which are rich in bioactive substances are attracting increasing attention. In this study, the nutritional value, chemical composition, biological activities, and feed indices of different parts of *Millettia speciosa* were comprehensively evaluated. In terms of its nutritional value, this study demonstrated that the leaves, flowers and seeds of *M. speciosa* were rich in elements and amino acids; the biological values (BVs) of these ingredients ranged from 85% to 100%, showing the extremely high nutritional value of this plant. GC-MS analysis suggested that the main chemical components of the flower volatile oil were *n*-hexadecanoic acid (21.73%), tetracosane (19.96%), and pentacosane (5.86%). The antibacterial activities of the flower and seed extracts were significantly stronger than those of the leaves and branches. The leaf extract displayed the strongest antifungal activities (EC_50_ values: 18.28 ± 0.54 μg/mL for *Pseudocryphonectria elaeocarpicola* and 568.21 ± 33.60 μg/mL for *Colletotrichum gloeosporioides*) and were the least toxic to mouse fibroblasts (L929) (IC_50_ value: 0.71 ± 0.04 mg/mL), while flowers were the most toxic (IC_50_ value: 0.27 ± 0.03 mg/mL). In addition, the abundance of fiber, protein, mineral elements, and functional metabolite contents indicated the potential applicability of *M. speciosa* as an animal feed. In conclusion, as a traditional herbal plant used for medicinal and food purposes, *M. speciosa* shows potential for safe and multifunctional development.

## 1. Introduction

Currently, the demand for a healthy and natural diet is increasing, and traditional plants that are rich in bioactive substances and are highly nutritious are attracting much attention [1]. *Millettia speciosa* Champ. is a leguminous herb that is mainly distributed in tropical and subtropical regions and is extensively cultivated in southeastern China. This herb is well known for its stamina-enhancing medicinal properties and high nutritious value, especially in the food and pharmaceutical industries [2]. As a type of folk medicine, *M. speciosa* is effective in the treatment of many chronic diseases, such as wrist numbness, diabetes, lumbago, rheumatoid arthritis, and chronic hepatitis [3]. This plant is rich in active molecules such as polysaccharides, flavonoids, alkaloids, and terpenoids, which possess antioxidant, antibacterial, antitumor, anti-inflammatory, hypolipidemic, and immunological activities [4,5].

Numerous studies have demonstrated the potential developmental value of the root of *M. speciosa*, which is rich in polysaccharides, flavonoids, and alkaloid metabolites with good antifatigue effects [6] and the ability to alleviate glycolipid disorder [7]. Additionally, an active substance present in the roots, MSCP2, promotes phagocytosis and stimulates cytokine or NO production [8]. It is also worth noting that the leaves are rich in bioactive plant proteins and can be used as a high-quality natural protein source [9]. However, less attention has been given to other parts of this plant, such as flowers, leaves, seeds or branches, and more research should focus on the nutritional value and bioactivities of the different tissues of *M. speciosa*.

As a traditional herbal plant used for both medicinal and food purposes, *M. speciosa* has been used for several practical applications and has been used to develop antioxidants in recent years; in contrast, the chemical fractions, such as volatile oils, elements, and amino acids, of *M. speciosa* are less well studied. Thus, obtaining information on the composition and biological activity of its chemical fractions is essential for the development and utilization of *M. speciosa* [10].

In this study, volatile oil was extracted from *M. speciosa* flowers and the chemical composition of the volatile oil was analyzed. This study evaluated the nutritional value indices of amino acids, elements, and fatty acids in *M. speciosa* leaves, branches, and seeds; performed high-performance liquid-phase analysis of secondary metabolites in leaves, branches, and seed pods; and further evaluated the inhibitory activities of these different tissue extracts against pathogenic agroforestry bacteria, fungi, and mouse fibroblasts (L929). Finally, the animal feed indicators of the leaf fractions of *M. speciosa* were evaluated. This study provides an important theoretical basis for the comprehensive development and utilization of *M. speciosa* resources.

## 2. Results

### 2.1. Minerals and Trace Element Composition

In this study, the types and contents of elements in the flowers, leaves, and seeds of *M. speciosa* were determined using ICP-MS, and the results are shown in Table 1. Twenty-eight elements were analyzed and detected in the flowers, leaves, and seeds of *M. speciosa*, among which K, Ca, Mg, and Na were abundant in the different parts. K was the most abundant element in the flowers, leaves, and seeds, with contents of 10,642 mg/kg, 9547 mg/kg, and 7919 mg/kg, respectively. The contents of Ca and Mg in the flowers were 1436 mg/kg and 1101 mg/kg, respectively, and the contents of Ca and Mg in the leaves were 3598 mg/kg and 1417 mg/kg, respectively. Fe, Zn, Cu, Cr, Mo, Se, and Co were the essential trace elements detected in the flowers, leaves, and seeds, with high levels of Fe, Zn, and Cu observed in all these tissues (flowers: Fe, 78 mg/kg, Zn, 19 mg/kg, Cu, 5.3 mg/kg; leaves: Fe, 106 mg/kg, Zn, 26 mg/kg, Cu, 5.7 mg/kg; and seeds: Fe, 34 mg/kg, Zn, 38 mg/kg, Cu, 7.1 mg/kg). The trace element Mn was a potentially available resource, with contents of 86 mg/kg, 137 mg/kg, and 37 mg/kg in the flowers, leaves, and seeds, respectively. The contents of elemental Pb in the flowers, leaves, and seeds were 0.48 mg/kg, 0.47 mg/kg, and <0.1 mg/kg, respectively, which was less than the limits specified in the standard (DBS 44-016/2021) (≤2.0 mg/kg) requirements, consistent with the production standards.

### 2.2. Amino acid Composition

**Figure 1:**
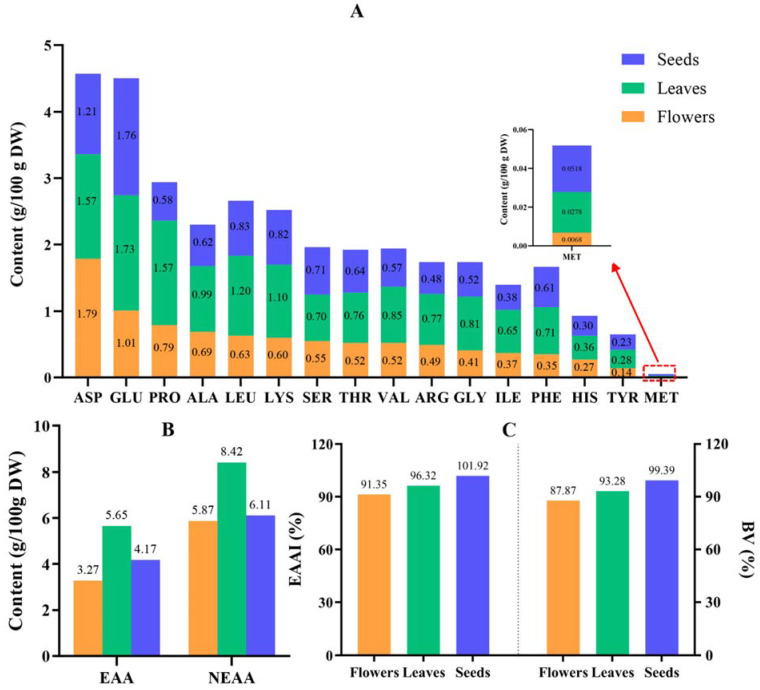
Amino acid content in different parts of *M. speciosa.* (**A**) Type and content of all amino acids in different tissues. (**B**) Content of essential and nonessential amino acids in different tissues. EAAs are essential amino acids and NEAAs are nonessential amino acids. (**C**) EAAI and BV in different tissues. EAAI is the essential amino acid index, and BV is the biological value.

The determined composition and content of amino acids in the flowers, leaves, and seeds of *M. speciosa* are shown in Figure 1. Aspartic acid (ASP) and glutamic acid (GLU) were the amino acids present at high levels in the leaves, flowers, and seeds, as shown in Figure 1A. In addition to ASP and GLU, proline PRO, LEU, LYS, and alanine (ALA) were present at high levels in the leaves. With 1.00 g/100 g applied as a limit, five amino acids exceeded this limit in the leaves, being present at 7.17 g/100 g, while two amino acids exceeded the limit in the flowers (2.80 g/100 g) and seeds (2.97 g/100 g). When 0.50 g/100 g was used as a limit, thirteen amino acids exceeded this standard in the leaves, with a content of 13.44 g/100 g, eleven in the seeds (8.87 g/100 g), and the fewest (only nine) in the flowers (7.10 g/100 g). The total content of the 16 amino acids detected in the leaves was the highest, at 14.07 g/100 g, followed by that in the seeds (10.28 g/100 g) and the flowers (9.14 g/100 g). Furthermore, Figure 1B shows that the leaves possessed the highest content of essential and nonessential amino acids, with values of 5.65 g/100 g and 8.42 g/100 g, respectively, which accounted for 40.16% (EAAs) and 59.84% (NEAAs) of the total amino acid content. As shown in Figure 1C, the EAAI and BV indices were different among the different parts of *M. speciosa.* The highest EAAI (101.92%) and BV (99.39%) indices were found in the seeds, followed by the leaves (EAAI, 96.32%; BV, 93.28%), and the lowest indices were found in the flowers (EAAI, 91.35%; BV, 87.87%). These results indicated that different parts of *M. speciosa* were rich in amino acids and had an extremely high nutritional value. The parts with the highest amino acid content were the leaves, followed by the seeds and flowers, while the nutritional value of the leaves and seeds was higher than that of the flowers.

### 2.3. Fatty Acid Composition

A total of 21 fatty acid components (Table 2) were detected in the seeds of *M. speciosa*, with the top three being linoleic acid (C18:2, 41.9%), oleic acid (C18:1, 27.3%), and palmitic acid (C16:0, 21.3%). In addition, stearic acid (C18:0, 4.99%) and docosanoic acid (C22:0, 1.16%) were present at more than 1%. Lauric acid (C12:0), pentadecanoic acid (C15:0), eicosadienoic acid (C20:2), docosadienoic acid (C22:2), timnodonic acid (C20:5), and docosahexaenoic acid (C22:6) were present at less than 0.05%. Saturated fatty acid content accounted for 29.27% of all fatty acid components; the ratio of UFAs to SFAs was 2.42, the *n*-6/*n*-3 and *n*-3/*n*-6 ratios were 48.95 and 0.02, respectively, and the AI and TI values were 0.31 and 0.71, respectively.

### 2.4. Feasibility as Animal Feed

To evaluate the potential of *M. speciosa* leafy branches as an animal feed, the basic components, functional metabolites, and minerals, including total phosphorus, in the leafy branches and their contents were further determined. The basic components and their contents in the leafy branches of *M. speciosa* are provided in Table 3. Among the basal nutrients, the most abundant were NDF (60.48%), ADF (49.29%), and acid detergent lignin (29.02%). In addition, the leafy branches were rich in crude protein, WSCs, and crude fat, and the crude ash content was 4.40%. Different functional metabolites were also observed in the leafy branches of *M. speciosa*, in which the contents of total phenols and total flavonoids were 6.09% and 4.81%, respectively. Among phenolic compounds, hydrolyzed tannins exhibited the highest content, at 4.17%, followed by simple phenols (1.93%), while condensed tannins had the lowest content (0.31%). The results obtained from the element analysis (Table 1 and Table 3) demonstrated that the contents of total phosphorus, K, Ca, and Mg in the leaves or leafy branches of *M. speciosa* were 0.14%, 9547 mg/kg, 3598 mg/kg, and 1417 mg/kg, respectively. In addition, the mineral elements with relatively high contents were Mn (137 mg/kg), Fe (106 mg/kg), Na (31 mg/kg), and Zn (26 mg/kg); information on the remaining mineral elements is shown in Table 1.

### 2.5. Chemical Composition of the Volatile Oil

A total of 54.5 mg of *M. speciosa* volatile oil was obtained using hydrodistillation, with a fresh weight yield of 0.03985‰ (based on fresh mass). Thirty-seven chemical components were identified from the volatile oil of *M. speciosa*, accounting for 67.54% of the total (Table 4). The components with high relative percentages were *n*-hexadecanoic acid (21.73%), tetracosane (19.96%), pentacosane (5.86%), and nonacosane (5.16%). The sum of the relative percentages was 63.81%, while the relative percentages of other components were less than 4%. 

### 2.6. Antibacterial Activity

**Figure 2:**
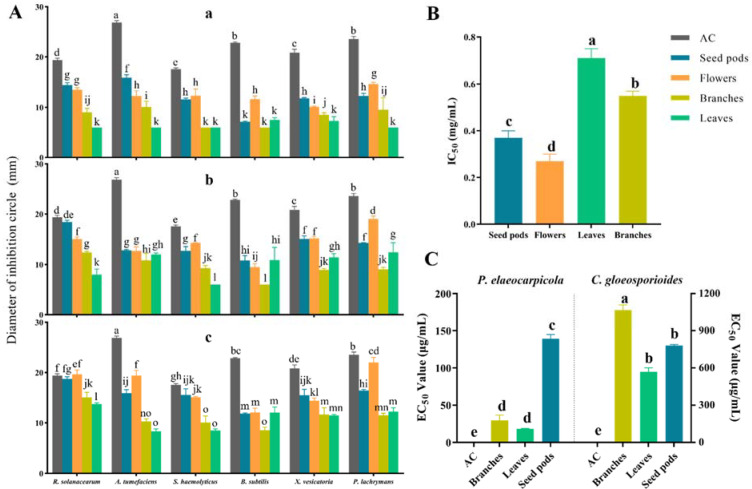
Biological activities of extracts from different parts of *M. speciosa*. (**A**), Antibacterial activity, (**a**), test sample concentration 30 mg/mL, (**b**), test sample concentration 60 mg/mL, (**c**), test sample concentration 90 mg/mL. AC indicates the positive control, streptomycin sulfate, all at 10 mg/mL. (**B**), Cytotoxic activity. (**C**), Antifungal activity. AC indicates the positive control, carbendazim. Different lowercase letters in the graph indicate significant differences (Duncan’s test, *p* < 0.05).

The inhibitory activities of different extracts from the seed pods, flowers, branches, and leaves of *M. speciosa* against the six test bacteria are shown in Figure 2A and Tables S2–S4. The results indicated that extracts from the different tissues of *M. speciosa* exhibited different degrees of antibacterial activity against different test bacteria; except for the seed pod and flower extracts, which showed no significant difference in inhibitory effect in comparison with that of a medium or high concentration of streptomycin sulfate, the inhibitory effect of the rest of the extracts was significantly lower than that of the positive control, streptomycin sulfate (AC). Moreover, the antibacterial activity of the different extracts against the different test bacteria varied at different concentrations, but in general, extracts at higher concentrations showed stronger antibacterial activity, suggesting a concentration effect. Among them, the seed pod and flower extracts exhibited stronger antimicrobial activity, showing inhibitory activity against all the tested bacteria at 30, 60, and 90 mg/mL concentrations. The antibacterial activity of the branch and leaf extracts was weaker. The branch extracts showed no inhibitory activity against *S. haemolyticus* at 30 mg/mL; however, they displayed inhibitory activity at both 60 and 90 mg/mL. The same phenomenon was observed for the inhibitory activity of the leaf extracts against *R. solanacearum*, *A. tumefaciens,* and *P. lachrymans*. The branch extracts exhibited no inhibitory activity against *B. subtilis* at 30 or 60 mg/mL, and the leaf extracts exhibited no inhibitory activity against *S. haemolyticus*.

### 2.7. Cytotoxic Activity

The cytotoxic activities of different extracts of *M. speciosa* on mouse fibroblasts (Figure 2B and Table S5) indicated that the ethyl acetate extracts of different tissues exhibited some inhibitory effect, and there were significant differences in IC_50_ values among the different tissues. The activity of the flower and seed pod extracts was significantly stronger than that of the leaves and branches in inhibiting mouse fibroblasts, and the flower extract exhibited the strongest cytotoxic activity, with an IC_50_ value of 0.27 ± 0.03 mg/mL; in contrast, the leaf extract showed the weakest cytotoxic activity, with an IC_50_ value of 0.71 ± 0.04 mg/mL. The above results suggested that the components of *M. speciosa* that exhibited inhibitory effects on mouse fibroblasts were mainly distributed in the flowers and seed pods.

### 2.8. Antifungal Activity

The inhibitory activities of extracts from the branches, leaves, and seed pods of *M. speciosa* against *P. elaeocarpicola* and *C. gloeosporioides* are presented in Figure 2C and Table S6. The inhibitory activities of extracts from different tissue parts of the plant against the two fungi differed significantly. Inhibitory activity against *P. elaeocarpicola* was significantly better than that against *C. gloeosporioides*; the EC_50_ values of extracts from all tissues against *C. gloeosporioides* were greater than 500 μg/mL, while the EC_50_ values of extracts from all tissues against *P. elaeocarpicola* were less than 200 μg/mL. The order of inhibitory activity against *P. elaeocarpicola* was leaf > branch > seed pod, while the order of inhibitory activity against *C. gloeosporioides* was leaf > seed pod > branch, which showed that the leaf extracts had the strongest inhibitory activity against both fungi, with EC_50_ values of 18.28 ± 0.54 μg/mL (*P. elaeocarpicola*) and 568.21 ± 33.60 μg/mL (*C. gloeosporioides*). 

## 3. Discussion

As a traditional medicinal and food plant with high nutritional value and diverse bioactivities, *M. speciosa* has the potential to be incorporated into nutritional health products and byproducts with high added value. The present study focused on evaluating the elements, amino acids, fatty acids, feed value indices, volatile oil composition, and multiple bioactivities of *M. speciosa*. 

Trace elements play a significant role in metabolism in humans, animals, and plants and play an important role in cell function and the maintenance of metabolic stability [15]. In this study, 28 elements were identified in different organs of *M. speciosa* using ICP-MS semiquantitative analysis. The investigation conducted in this study also demonstrates that mineral element content varies among the different parts of the plant. Among them, K was the most abundant element in different tissues, and the plant tissues with the highest to lowest K content were flowers, leaves, and seeds. K is among the bulk nutrients necessary for plant growth and plays an important role in photosynthesis, photosynthetic product translocation, stomatal movement regulation, phloem transport, crop yield, quality, and stress resistance [16]. The elements Ca and Mg were also abundant in different parts, with seeds having the highest content of Mg (2074 mg/kg) and leaves having the highest content of Ca (3598 mg/kg), followed by flowers (1436 mg/kg) and leaves (925 mg/kg). The content of seven essential trace elements (Fe, Zn, Cu, Cr, Mo, Se, Co), other than elemental iodine (I), was the highest in leaves, followed by flowers and seeds. Among these essential trace elements, the contents of Fe, Zn, and Cu were relatively high. In combination with other elements such as Cr, Mo, and Co, these elements play a substantial role in cell function and the maintenance of metabolic stability [15]. Fe content in flowers (78 mg/kg) and leaves (106 mg/kg) was higher than that in beef liver (58 mg/kg) [17]. Se is an essential trace element in the human body that can slow aging, enhance immunity, and prevent cardiovascular diseases [18]; the main source of Se is food, such as nuts, cereals, poultry, fish, or seafood. Ingesting excess or insufficient levels of Se can pose various hazards to the human body; the optimal range for Se concentration in plasma is 90~120 µg/L. The seeds and leaves of *M. speciosa* were rich in Se, with 0.59 mg/kg in the seeds and 0.24 mg/kg in the leaves, which is higher than the levels in traditional foods, such as 0.35~0.47 mg/kg in beef, 0.4 mg/kg in lamb, 0.21~0.27 mg/kg in salmon, 0.17 mg/kg in eggs, and 0.1~0.55 mg/kg in various dairy products [19]. As a mineral element essential for normal growth and bone development in all organisms, phosphorus plays an important role in a variety of metabolic processes, such as carbohydrate and lipid metabolism [20]. Total phosphorus is an essential nutrient indicator in the diets of aquatic and terrestrial animals, such as fish and pigs [20,21]. The total phosphorus content in the leaves of *M. speciosa*, at 0.14%, was lower than the reference value of 0.5~1% for total phosphorus content in feeds [22]. Evaluating the micronutrients in *M. speciosa* is important for finding alternative foods or animal feed sources. However, not all trace elements are beneficial to organisms; Pb, Cd, As, and Hg are trace elements that are harmful and exhibit high toxicity. The contents of Pb, Cd, Hg, and As in *M. speciosa* all corresponded with the current Guangdong Province Food Safety Local Standard (DBS 44-016/2021) of China (Pb ≤ 2 mg/kg) and “the National Standard for Food Safety Limits of Contaminants in Food” standard [23] for harmful elements (Pb ≤ 5 mg/kg, Cd ≤ 1 mg/kg, As ≤ 2 mg/kg, Hg ≤ 0.2 mg/kg). Therefore, in terms of these trace elements, *M. speciosa* is safe and meets the production requirements.

Amino acids are nontoxic and nonpharmacological general nutrients that play an important role in the physiology and reproduction of living cells. Studies on amino acids present in various plants have been reported [24]. In this study, the types and contents of sixteen amino acids, including eight essential amino acids and eight nonessential amino acids, were determined in *M. speciosa*, providing useful information for further exploitation of the plant. The results demonstrated that the leaves (14.1 g/100 g) possessed the highest total amino acid content, followed by the seeds (10.3 g/100 g) and flowers (9.14 g/100 g). This result also supported results from previous studies showing that most of the amino acids were produced in the root and leaf parts of the plant [25]. The content of essential amino acids can be used as an indicator of the quality of dietary proteins [26] and is an important nutritional indicator used to evaluate food or feed. The leaves exhibited the highest content of essential amino acids (5.65 g/100 g, 40.07%), which was close to that of alfalfa, a traditional forage plant (43.5%) [27]. Notably, the proportion of essential amino acids to total amino acids in all parts exceeded that in powdered milk by 35%, showing an extremely high nutritional value [28]. The proportion of EAAs to total amino acids in the leaves (40.07%) and seeds (40.49%) was close to the previously recommended value of 40% [29]. GLU, PRO, and LEU are functional amino acids that are obtained via the diet and participate in metabolic regulation to protect the health of the organism [30]. Previous studies have shown that SER may stimulate the proliferation of cancer cells [31]. In the present study, SER contents in the flowers, leaves, and seeds were 0.55, 0.70, and 0.71 g/100 g, respectively, which were lower than the reference values [29]. LEU and LYS are important essential amino acids in animal feeds, but most currently used plant-based feeds are deficient in LEU and LYS [27]. The LEU and LYS contents in the leaves and seeds were lower than those in soybean flour (LEU, 2.46 g/100 g; LYS, 2.0 g/100 g); however, all of these levels were higher than those in maize flour, rice flour, and wheat flour, indicating that *M. speciosa* is a high-quality source of LEU and LYS [32]. The ratio of all essential amino acids in a target protein to a high-nutritional value reference is known as the EAAI index [33]. In this study, the EAAI index of flowers (91.35%), leaves (96.32%), and seeds (101.92%) derived from the whole plant as a reference was distributed in the range of 90~102%, displaying an extremely high nutritional value. The target protein quality was excellent when the BV index was within 70~100% [34]. In this study, the BV values of *M. speciosa* flowers, leaves, and seeds ranged from 85% to 100%, indicating that *M. speciosa* has potential for the development of high-quality foods.

This study indicated that the seeds of *M. speciosa* possessed favorable lipid nutritional value. Fatty acids are among the main components of lipids and can participate in various important metabolic pathways in organisms and improve the ability of organisms to interfere with external biotic and abiotic factors [35]. Fatty acids from plants are an excellent source of nutrition for both humans and animals, and the intake of certain fatty acids is closely related to human health; for instance, unsaturated fatty acid intake can reduce the risk of cancer. Fatty acids can be developed as natural and environmentally friendly antifungal agents and good food additives due to their safety and bioactivity [36]. Unsaturated fatty acids exhibit stronger biological activity than saturated fatty acids, which may be related to the double-bond structure in unsaturated fatty acids, which can increase the freedom of movement of biofilms [37]. The seeds of *M. speciosa* exhibited a higher content of unsaturated fatty acids (70.82%) than saturated fatty acids (29.27%), meaning that the seeds may possess stronger bioactivities of highly abundant fatty acids that are beneficial to humans or animals and that they are the simplest and safest source of fatty acids for ruminants, especially of linoleic or linolenic acid, which account for a high percentage of the total [38]. Although the content of linolenic acid in *M. speciosa* was relatively low (0.807%), linoleic acid accounted for 41.9% of the total fatty acids and was the most abundant fatty acid, followed by oleic acid (27.3%) and palmitic acid (21.3%). Linoleic and oleic acids are unsaturated fatty acids that inhibit the growth of the mycelia of pathogenic fungi (*Rhizoctonia solani*, *Pythium ultimum*, *Pyrenophora avenae,* and *Crinipellis perniciosa*) at a concentration of 1000 µM [39]. Palmitic acid is recognized as a safe substance in food with minimal risk to humans and has a wide range of uses within the field of care products and cosmetics. Palmitic acid is a saturated fatty acid that reduces the fluidity of liver cancer cell membranes, regulates glucose metabolism, and exhibits some antibacterial, antioxidant, and insecticidal activities [40,41]. In addition, palmitic acid (21.3%) content was on par with or better than that of some high-quality forages, such as Jerusalem artichoke herbage (22~26%), alfalfa (15~30%), perennial ryegrass (*Lolium perennne* L., 16~20%), red clover (14~20%), and corn silage (16%) [42]. The UFA/SFA value of 2.42 found in *M. speciosa* seeds was higher than the recommended value of 0.35, indicating its high nutritional quality [43]. Atherosclerosis index (AI) and thrombosis index (TI) indicate the potential to stimulate platelet agglutination; the lower the value is, the greater the potential for protection against coronary artery disease, with recommended values of 1 (AI) and 0.5 (TI), respectively [44]. In this study, the AI value was 0.31, which is below the recommended value; in contrast, the TI value (0.70) exceeded 0.5. The *n*-6/*n*-3 ratio in the diet is closely related to various human diseases, and the published value for the prevention of cardiovascular risk is below 4.00% [45]. In this study, this ratio reached 48.95%, suggesting that *M. speciosa* seeds may not be suitable for routine direct consumption. The rich composition and content of fatty acids in the seeds of *M. speciosa* demonstrated the nutritional value and exploitation potential of this plant. 

Plant-based feed resources have an important impact on agriculture and animal husbandry because these resources are rich in nutrients such as fiber, protein, mineral elements, and fatty acids [42]. Analyses of the previously discussed nutritional indicators of micronutrients, amino acids, and fatty acids have demonstrated that *M. speciosa* has the potential to be developed as a plant-based feed. Hay forage is an important plant-based feed resource, and high-quality hay forage can help maintain the normal feeding behavior and nutritional requirements of animals [46]. To evaluate the potential of *M. speciosa* leafy branches as a hay feed, the present study was carried out to evaluate the levels of crude protein, WSC, NDF, ADF, crude fat, crude ash, and ADL on the basis of the dry weight of *M. speciosa* leaves. Crude protein content was 14.2%, which is higher than that in the commonly used plant-based feed, maize leaves (11.4%), and lower than that in alfalfa leaves (15.3% to 25.8%) [34,47]. The content of crude fat (1.4%) was close to that in maize leaves (2%) and alfalfa leaves (2.4% to 3.8%) [47,48,49]. Crude ash content was 4.4%, which was in the normal range [15]. WSC, NDF, and ADF levels are important indicators for evaluating plants as feedstuffs, especially WSCs, which are crucial for energy metabolism in ruminants, and a higher WSC content leads to higher digestibility of feed. In this study, the NDF value in *M. speciosa* leafy branches (60.48%) was higher than that of alfalfa hay (44.01%) and corn silage (42.07%), although not as high as that of corn stover (73.65%), rice straw (71.48%), and straw (81.47%), whereas the ADF value (49.29%) of *M. speciosa* leafy branches was close to that of straw (51.42%) and was higher than that of corn stover (43.13%), rice straw (45.9%), alfalfa hay (31.61%), and corn silage (24.62%) [50]. Hence, it is evident that there were abundant cellulose resources in the leafy branches of leafy branches. The WSC content of *M. speciosa* leafy branches was relatively low, at 5.82%, which was lower than that in corn stover feedstock (12%) [51], which may be related to the high content of NDF (60.48%) and the season or time of collection [52]. 

Essential oils (EOs), terpenoids, polyphenols, and flavonoids are important phytochemicals that play an important role in improving feed utilization and animal health [53]. Additionally, many studies have shown that phenolic and flavonoid metabolites are essential for humans and are closely related to a variety of biological activities, such as antioxidant, antimicrobial, anticancer, hepatoprotective, and cardioprotective activities [54,55]. Therefore, evaluating secondary metabolites and their multiple biological activities from different tissues of *M. speciosa* is particularly necessary. Functional metabolite results presented high total phenolic and total flavonoid contents of 6.09% and 4.81%, respectively, in the leafy branches of *M. speciosa*. Total phenols are naturally present in plant material and the appropriate addition of phenolic compounds to animal feed can increase animal performance. However, added content should not be as high as possible; rather, it needs to be within a certain suitable range. Based on the reports of Levickienė and Yang et al. on plants with the potential to be developed for food or feeding, it can be concluded that the total phenolic and total flavonoid contents of *M. speciosa* leafy branches were in the suitable range of contents (total phenols, 5.32–9.15%; total flavonoids in Lithuanian mulberry leaves, 9.22–15.12%). Meanwhile, the total flavonoid content was lower than that of another medicinal and food plant, *Zingiber striolatum* (>15%) [56,57,58].

In this regard, phenolic compounds were mainly hydrolyzed tannins (4.17%) and simple tannins (1.93%), with the lowest content observed for condensed tannins (0.31%), indicating a similar potential for development as that of *Moringa* [26]. The main chemical components of the flower volatile oil were *n*-hexadecanoic acid (21.73%), tetracosane (19.96%), and pentacosane (5.86%). Plant volatile oils contain simple compounds with low molecular weights and volatile aromatic properties; they are secondary plant metabolites with various biological activities, such as antibacterial, antioxidant, anti-inflammatory, antiviral, and antitumor activities, and the biological activities of plant volatile oils are often inextricably linked to their chemical composition [54]. Previous studies have shown that *n*-hexadecanoic acid demonstrates excellent antioxidant and antibacterial activities [59]. Since the yield of the volatile oil from *M. speciosa* flowers in this study was only 0.03985‰ (based on fresh mass), volatile-oil-related activities were not examined. However, the biological activity of the volatile oil of *M. speciosa* flowers should be further investigated in the future.

Plant secondary metabolites and their derivatives are important resources for the development of novel, naturally active medicines [60]. Various studies have suggested that extracts of *M. speciosa* exhibit strong antioxidant activity, and a variety of active molecules have been obtained from them [61]. However, the biological activities of the plant extracts remain understudied, and the present study investigated the antibacterial and antifungal activities of different parts of *M. speciosa* by using common pathogenic bacteria from agroforestry as test targets. The extracts of *M. speciosa* exhibited significantly stronger antifungal activity against *P. elaeocarpicola* than that of *C. gloeosporioides*. The leaf extracts exhibited the strongest antifungal activity, followed by the branches and the seed pods. The seed pod and flower extracts exhibited significantly stronger antibacterial activity against six tested bacteria (*R. solanacearum*, *A. tumefaciens*, *X. vesicatoria*, *P. lachrymans*, *E. coli*, *S. haemolyticus* and *B. subtilis*) and exhibited stronger inhibitory activity than that of the leaf or branch extracts. For cytotoxic activity analysis, mouse fibroblasts (L929) were selected as the test subjects. L929 cells are nontumor cells that are widely used in testing the cytotoxicity of plant materials due to their highly sensitive and reproducible response to toxicity [62]. Sforcin and Campoccia et al. concluded that in evaluating plant crude extracts for toxicity to human cancer cells or normal cells (fibroblasts L929), the IC_50_ value should be less than 30 μg/mL, or less than 25 μg/mL under strict conditions [62,63]. The results of cytotoxic activity analysis performed in this study showed that the IC_50_ values of flower, seed, and leaf extracts from the aboveground parts of *M. speciosa* were all higher than 25 μg/mL against L929 cells, indicating that the aboveground parts of the plant were weakly toxic to normal cells. The flower and seed extracts had significantly stronger inhibitory activity than the leaf and branch extracts, while the leaf extracts presented the highest IC_50_ values (0.71 ± 0.04 mg/mL) against fibroblasts (L929), showing the lowest toxicity. The magnitude of the toxicity of different parts of the plant must be considered in future studies of anticancer agents from *M. speciosa*. In the future, the chemical structure and pharmacological activities of the active compounds in *M. speciosa* must be studied. Furthermore, research on germplasm preservation and breeding techniques should be accelerated to provide a scientific basis for elucidating the mechanism of action and developing and utilizing *M. speciosa* resources.

## 4. Conclusions

In this study, a comprehensive evaluation of the nutritional value, chemical composition, bioactivities, and feed indicators of different tissues of *M. speciosa* was carried out. The results obtained from the nutritional value evaluation indicated that the flowers, leaves, seeds, and branches of *M. speciosa* were rich in elements, amino acids, and fatty acid resources (seeds), and their BVs ranged from 85% to 100%, revealing an extremely high nutritional value, indicating that it might perhaps serve as a good food source. The main components of the flower volatile oil were *n*-hexadecanoic acid, tetracosane, and pentacosane. Secondary metabolites were also abundant in the leaf, branch, and seed pod extracts and exhibited good antibacterial, antifungal, and cytotoxic activities. In particular, the flower and branch extracts showed stronger antibacterial and cytotoxic activity. The leaf extract exhibited the strongest antifungal activity and the weakest inhibitory activity against murine fibroblasts (L929). Moreover, the abundance of fiber, protein, amino acid, mineral element, and functional metabolite contents also suggest that *M. speciosa* has the potential to be developed as an animal feed. In conclusion, as a traditional herbal plant with both medicinal and food uses, *M. speciosa* can be developed for safe and multifunctional applications.

## 5. Materials and Methods

### 5.1. Plant Materials

The flowers, leaves, branches, seeds, seed pods, and leafy branches (branches with leaves attached) of *M. speciosa* were collected from the planting base of a *M. speciosa* plant grown at Guangdong Nature Lover Agriculture Technology Limited Company, Jiangmen, Guangdong, China. Pictures of the plant’s planting panorama and tissue parts are visible in Supplementary Material Figure S1. The samples were transported to the laboratory using aseptic self-sealing bags and immediately transferred to storage at 4 °C for subsequent use.

### 5.2. Trace and Mineral Elements

The elements present in flowers, leaves, and seeds were analyzed using a semiquantitative inductively coupled plasma–mass spectrometry (ICP-MS) method [64]. Different samples were first weighed out (50 g), dried, powdered in a high-speed pulverizer, and sieved with a 150 μm sieve. Then, 0.20 g of the sample was accurately weighed out and acid (HNO_3_) digestion was performed. The sample was ventilated at 160 °C for 60 min, and water was used to adjust the residual solution volume to 50 mL. Finally, identification was completed by an ICP-MS instrument, with the determination procedure described in the “National Standard for Food Safety—Determination of Multiple Elements in Food” [65]. Total phosphorus content was measured using methods previously described in the literature, with some improvements [20]. Briefly, leaf samples (2.0 g) were dried and ashed at 550 °C for 3 h. Following ashing, the samples were digested with hydrochloric acid and nitric acid at 100 °C for 10 min. The digestion solution was filtered after cooling and subsequently diluted with distilled water to 100 mL. The diluted solution was mixed thoroughly with vanadium molybdate reagent and cooled at room temperature for 10 min. Finally, it was analyzed at 400 nm using a spectrophotometer (T-N5000, Shanghai, China).

### 5.3. Amino Acids

The composition and content of amino acids were determined with reference to the National Standard for Food Safety of the People’s Republic of China [66]; 16 acid-hydrolyzed amino acids were measured in the flowers and leaves of *M. speciosa* using an amino acid analyzer (ninhydrin postcolumn derivatization ion exchange chromatography). Briefly, 2.0 g each of leaves, flowers, and seeds were weighed separately and mixed with 10 mL of hydrolysis solution (6 mol/L HCl, 0.1% phenol) in a 10 mL airtight glass vial tightly closed under nitrogen (Hangzhou Mio Instruments Co.). Following hydrolysis at 110 °C for 22 h, the solution was filtered and diluted to 50 mL at room temperature. One milliliter of the solution was placed in a test tube under reduced pressure and then dissolved using 2 mL of water, followed by drying under reduced pressure. Subsequently, the solution was fully dissolved using 2 mL of sodium citrate buffer (pH 2.2), filtered (0.22 μm organic membrane), and transferred to an injection bottle for testing. The configuration steps performed for the mixed amino acid standard reserve solution (1 μmol/mL) and standard working solution (100 nmol/mL) were as follows: The standard amino acid samples were weighed out accurately (Table S1) and fully dissolved in 8.3 mL of 6 mol/L HCl solution. The volume was adjusted with water to 250 mL, and the sample was then mixed thoroughly. Then, 1 mL of the solution was aspirated into in a 10 mL volumetric flask and adjusted with sodium citrate buffer (pH 2.2) to prepare a mixed amino acid standard working solution (100 nmol/L). Similar volumes of mixed amino acid standard working solution and test samples were tested on the instrument (column: sulfonic acid-type cation resin; detection wavelengths: 570 nm and 440 nm). Finally, the peak area was used to calculate the concentration of amino acids in the determined samples by the external standard method.

In this study, whole egg protein was used as a reference standard [67] to calculate the essential amino acid index (EAAI) according to a previously described method [68]. Finally, the biological value (BV) was derived from the following equation [34]:

(1) $$\mathrm{EAAI}\;(\%)=\sqrt[{n}]{\prod_{i=1}^{n}\left(aai/AAi\right)}$$

BV (%) = 1.09 × EAAI − 11.7(2)

where *aa*_i_ is the percentage of an essential amino acid in the total essential amino acids in the *M. speciosa* experimental group, *AA*_i_ is the percentage of the corresponding essential amino acids in the reference protein to the total essential amino acids, and n is the number of essential amino acids included in the calculation.

### 5.4. Fatty Acids

The fatty acid content of *M. speciosa* seeds was determined according to the “Normalization method” in the “National standard for food safety—Determination of fatty acids in food” Chinese national standard [69]. First, 10.0 g of seeds was accurately weighed out, crushed using a high-speed crusher, and placed in a 250 mL triangular flask together with 100 mg of pyrogallic acid (with a small amount of zeolite added). Then, 2 mL of ethanol (95%) and 10 mL of HCl solution (8.3 mol/L) were added. The sample was mixed thoroughly, hydrolyzed at 75 °C for 40 min in a water bath, and cooled to room temperature. Next, 10 mL of ethanol (95%) was continually added, and the sample was mixed well. Afterward, the solution was immediately transferred to a partition funnel and extracted using 50 mL of a mixture of ether and petroleum ether (1:1 *v*/*v*). The extract was collected and the procedure was repeated three times. Then, the collected extracts were concentrated to dryness under reduced pressure to obtain the fat extract, which was refrigerated at 4 °C and set aside. Eight milliliters of sodium hydroxide methanol solution (2%) was added to the fat extract; the flask was connected to a reflux condenser, and the sample was refluxed in a water bath at 80 °C until the oil droplets disappeared. Then, 7 mL of boron trifluoride methanol solution (15%) was added from the upper end of the reflux condenser, refluxing was continued in a water bath for 2 min, and the flask was removed and cooled to room temperature. After 20 mL of *n*-heptane was added to the treated fat extract and shaken for 2 min, saturated aqueous sodium chloride was added, and the sample was left to stand for 30 min. Subsequently, 5 g of anhydrous sodium sulfate was added to 5 mL of the supernatant, which was shaken for 1 min and then left to stand for 5 min. Finally, the supernatant was transferred to an injection bottle for measurement. 

The samples were analyzed by an Agilent 7890B GC-5977B MSD. The parameters of the capillary column were as follows: column length 100 m, inner diameter 0.25 mm, and film thickness 0.2 μm. The injector and detector temperatures were 270 °C and 280 °C, respectively, with the following ramp-up procedure: initial temperature 100 °C for 13 min; ramp-up from 100 °C to 180 °C for 6 min; ramp-up from 180 °C to 200 °C for 20 min; ramp-up from 200 °C to 230 °C for 10.5 min. The sample volume was 1.0 μL, and helium was used as the carrier gas, with a splitting ratio of 100:1.

The numbers of total essential fatty acids, monounsaturated fatty acids, and polyunsaturated fatty acids in this study were calculated as follows:

SFA = ∑ (C12:0, C14:0, C15:0, C16:0, C17:0, C18:0, C20:0, C21:0, C22:0, C23:0, C24:0)(3)

MUFA = ∑ (C16:1, C18:1, C20:1, C22:1)(4)

PUFA =∑ (C18:2, C18:3, C20:2, C22:2, C20:5, C22:6)(5)

Finally, the atherogenic index (AI) and the prothrombotic index (TI) were calculated as previously described [68].

### 5.5. Basic Components

The basic nutrient content in of dried powdered samples of *M. speciosa* leafy branches was analyzed. Ash content was determined from dry samples after burning (550 °C) for 6 h [70]. Crude protein was analyzed using the Kjeldahl method for the determination of nitrogen content (N × 6.25). The main steps of the method were sulfuric acid digestion, alkali (NaOH) distillation, and titration with standard hydrochloric acid solution under catalytic conditions [69]. Crude fat was measured by the ANKOMXT10 filter bag method, which mainly consisted of adding 5.0 g of dry powder sample to an extraction tube and extracting with petroleum ether. After the solvent was removed by distillation and drying, the residue was dried to a constant weight in an oven, and the crude fat content was calculated [70]. The content of water-soluble carbohydrates (WSCs) was measured using the anthrone colorimetric method. Cellulose composition, neutral detergent fiber (NDF), acid detergent fiber (ADF), and acid detergent lignin (ADL) were assayed using Van’s fiber assay [71]. After 0.5 g of dry powder was weighed into the weighed filter bag, which was sealed at 4 mm from the bag opening, the bagged holder was subsequently placed into the fiber analyzer container with a blank filter bag for calibration and the sample was measured using an ANKOM2000i automatic fiber meter. After analysis and washing, the drained filter bag was loaded into a 250 mL beaker and soaked in sufficient acetone for 5 min. Then, the bag was air-dried, placed in an oven for 2–4 h (105 °C), transferred to a desiccator to isolate the air, cooled, and weighed, followed by the calculation of fiber content (NDF and ADF). ADL content was calculated by soaking the sample in 72% sulfuric acid solution for 3 h after ADF was measured; the residual sulfuric acid was then washed off, and the sample was dried and weighed.

### 5.6. Functional Metabolites

Gallic acid was used as the standard for phenolic content determination, and the total phenol and simple phenol levels were determined by the Folin–Ciocalteu method [72]. The difference between the total phenol and simple phenol levels was hydrolyzed tannins. Subsequently, proanthocyanidin was used as the standard for condensed tannin content, which was determined after color development using hydrochloric acid–acetone–butanol solution extraction. Total flavonoid content was measured by using the AlCl_3_ colorimetric method with rutin as the standard [73]. 

### 5.7. Preparation of Volatile Oil and GC-MS Analysis

Essential oil was isolated from fresh flowers by water vapor distillation using a Clevenger-type apparatus, and the specific procedure was as follows: A total of 1400 g of fresh flowers was packed into a 5 L flask, and an appropriate amount of ultrapure water was added to submerge the plant material. Subsequently, the flask was heated in a heating jacket with a temperature gradient from 10 °C to 180 °C and distilled continuously for 10 h, and the evaporated volatile oil was collected at the same time. Immediately after a certain amount of NaCl was added, the mixture was extracted three times using anhydrous ether, and the extract was dried to obtain the volatile oil from *M. speciosa* flowers, which was stored in a sealed container at 4 °C until further use.

The volatile oil of *M. speciosa* (50 μL) was first diluted with acetone (400 μL) and then filtered using a 0.22 μm organic filter membrane. The components of the volatile oil of *M. speciosa* were analyzed by an Agilent 7890B GC-5977B MSD. The capillary column was a DB-5 (30 m × 250 μm × 0.25 μm) with a split ratio of 1:1 and an injection volume of 1 μL. The ion source was EI (70 eV). The inlet and connection port temperatures were 230 °C and 250 °C, respectively. The column temperature chamber ramp-up procedure was as follows: 50 °C (held for 5 min), 5 °C/min to 210 °C (held for 2 min), and 25 °C/min to 260 °C (held for 2 min). The carrier gas was helium at a flow rate of 1 mL/min. The components of the volatile oil were identified by comparing their RIs (calculated using C_8_-C_40_) with the RI values of standard compounds in the NIST (2014) database and the characteristic peaks in the mass spectra. The relative amount of each component in the essential oil from the flowers was expressed as a percentage (%) of the total peak area.

### 5.8. Extraction of Nonvolatile Secondary Metabolites

Secondary metabolites from the leaves, seeds, and branches of *M. speciosa* were extracted by ethyl acetate at room temperature. Two kilograms of dried leaves, seeds, and branches were weighed out separately, crushed, placed in a clean glass container, and immersed in an appropriate amount of ethyl acetate at room temperature for 7 days. Each treatment was repeated three times. Finally, the extracts were filtered and concentrated to dryness under reduced pressure to obtain ethyl acetate extracts of different tissues of *M. speciosa*, which were subsequently stored at 4 °C and set aside.

### 5.9. Antibacterial Activity Evaluation

The antibacterial activity of different extracts of *M. speciosa* was evaluated based on the literature [74] with some improvements. The bacterial strains used were five Gram-negative strains (*Ralstonia solanacearum*, *Agrobacterium tumefaciens*, *Xanthomonas vesicatoria*, *Pseudomonas lachrymans* and *Escherichia coli*) and two Gram-positive strains (*Staphylococcus haemolyticus* and *Bacillus subtilis*). All the test strains were stored in the Laboratory of Plant and Microbial Health, College of Forestry and Landscape Architecture, South China Agricultural University. First, the test strains stored at −20 °C were inoculated onto LB solid medium and cultivated for 48 h. Afterward, single colonies were picked and incubated in LB liquid medium (28 °C, dark, 150 r/min) for 24 h. The concentration of the bacterial solution was adjusted to 10^8^ cfu/mL under aseptic conditions. Subsequently, 50 μL of the bacterial solution was added to LB solid plate medium using a pipette, and the plate was coated evenly. Small holes (6 mm in diameter) were punched in the plate using a sterile punch. Then, 40 μL of each sample being tested was added in each hole, and the plate was incubated for 24 h under dark conditions. The final concentrations of the test samples were set at 30, 60, and 90 mg/mL, while streptomycin sulfate (positive control) was set at 10 mg/mL. Each of the above treatments was repeated three times. Finally, the diameter of the inhibition zone was measured for subsequent statistical analysis.

### 5.10. Antifungal Activity Evaluation

The antifungal activity of different extracts of *M. speciosa* was investigated using the mycelial growth rate method [75,76,77]. The pathogenic fungi used for the test were *Pseudocryphonectria elaeocarpicola* and *Colletotrichum gloeosporioides*, which were stored in the Laboratory of Plant and Microbial Health, College of Forestry and Landscape Architecture, South China Agricultural University. First, eight different gradient concentrations of master mixes were prepared by completely dissolving different extracts using 30% DMSO solution. Second, one milliliter of the master mixes at different concentrations was injected into 29 mL of sterile PDA medium and evenly distributed into three plates to prepare toxic plates with final concentrations of 12, 8, 6, 4, 2, 1, 0.5, 0.25, and 0.125 mg/mL for subsequent experiments. Sterile water was used as a blank control, 30% DMSO solution was used as a negative control, and carbendazim was used as a positive control (final concentrations of 0.2, 0.1, 0.05, 0.025, 0.0125, 0.00625, and 0.003125 mg/mL). All of the above treatments and concentrations were repeated three times. Subsequently, cakes of the same size were punched out from the edge of the pathogenic fungi cultured for 7 days (25 °C, dark incubation) using a 0.7 cm diameter punch and inoculated onto the virulent plates. Finally, when the fungal hyphae of the blank control group grew to 2/3 of the medium area, the colony growth diameters of the different treatments were measured using the crossover method, and the following formula was used to calculate the inhibition rate:

(6) $$I=\frac{L_{1}-\;L_{2}}{L_{1}}\times \;100\%$$

where *I* indicates the inhibition rate, *L*_1_ indicates the pure growth of negative control colonies, and *L*_2_ indicates the pure growth of treated colonies.

### 5.11. Cytotoxicity Evaluation Assay 

The test of the toxic activity of different extracts of *M. speciosa* on cancer cells was based on a previously reported method with some modifications [78]. Different extracts were first prepared as 50, 100, 150, and 200 μg/mL test samples using DMEM (HyClone) cell culture medium with 5% (w) FBS (Gibco). After 200 μL of the test samples was added to each well of 96-well microplates, mouse fibroblast (L929) cell suspensions (density of 2 × 10^4^ cells/well) were inoculated into the microplates. All the treatments were replicated three times. The plates were subsequently incubated in a cell incubator for 24 h (37 °C, φ (CO_2_) 5%), with wells without samples included as blank controls. Immediately afterward, 15 μL of CCK-8 reagent was added into each well, and incubation was continued for 4 h (37 °C, φ (CO_2_) 5%). Finally, absorbance at 450 nm (D450 nm) was recorded for data evaluation using a multifunctional microplate detector at 450 nm. Mouse fibroblast (L929) cells were provided by the Affiliated Stomatology Hospital of Guangzhou Medical University.

### 5.12. Statistical Analysis

Data were recorded using Excel, plotted using GraphPad Prism (7.0), and statistically analyzed using SPSS (25.0), with all differences in data expressed as the mean ± standard deviation (SD). Student’s *t* test was used to compare pairs of groups. One-way ANOVA was performed for multigroup comparisons, and Duncan’s test was used to analyze significant differences in multiple comparisons. 

## Figures and Tables

**Table 1:** Elemental content in different parts of *M. speciosa*.

Element	Content (mg/kg)			Element	Content (mg/kg)		
	Flower	Leaves	Seeds		Flower	Leaves	Seeds
Li	<0.1	<0.1	<0.1	Ni	2.2	0.91	3.0
B	8.3	16	7.8	Cu	5.3	5.7	7.1
Na	22	31	<3	Zn	19	26	38
Mg	1101	1417	2074	As	<0.1	<0.1	<0.1
Al	31	54	<2	Se	<0.1	0.24	0.59
K	10,642	9547	7919	Rb	24	20	18
Ca	1436	3598	925	Sr	2.2	6.2	0.24
Ti	0.44	0.44	<0.1	Ag	<0.1	<0.1	<0.1
V	<0.1	<0.1	<0.1	Cd	<0.05	<0.05	<0.05
Cr	0.42	0.52	<0.1	Sn	<0.1	<0.1	<0.1
Mn	86	137	37	Sb	<0.1	<0.1	<0.1
Fe	78	106	34	Ba	4.3	8.4	0.42
Co	<0.1	0.10	<0.1	Hg	<0.05	<0.05	<0.05
Mo	0.37	<0.1	0.26	Pb	0.48	0.47	<0.1

**Table 2:** Fatty acid composition of *M. speciosa* seeds.

Fatty Acid	Percentage (%)	Fatty Acid	Percentage (%)
Lauric acid (C12:0)	0.0156	Erucic acid (C22:1)	0.0811
Myristic acid (C14:0)	0.130	Tricosoic acid (C23:0)	0.0998
Pentadecanoic acid (C15:0)	0.0121	Docosadienoic acid (C22:2)	0.0160
Palmitic acid (C16:0)	21.3	Timnodonic acid (C20:5)	0.0180
Palmitoleic acid (C16:1)	0.338	Tetracosanoic acid (C24:0)	0.798
Heptadecanoic acid (C17:0)	0.127	Docosahexaenoic acid (C22:6)	0.0309
Stearic acid (C18:0)	4.99	Total SFAs	29.27
Oleic acid (C18:1)	27.3	Total MUFAs	28.02
Linoleic acid (C18:2)	41.9	Total PUFAs	42.79
Linolenic acid (C18:3)	0.807	UFAs: SFAs	2.42
Arachidic acid (C20:0)	0.580	*n*-6/*n*-3	48.95
Eicosanoenoic acid (C20:1)	0.305	*n*-3/*n*-6	0.02
Heneicosanoic acid (C21:0)	0.0559	AI	0.31
Eicosadienoic acid (C20:2)	0.0230	TI	0.70
Docosanoic acid (C22:0)	1.16		

Note: SFAs, saturated fatty acids; MUFAs, monounsaturated fatty acids; PUFAs, polyunsaturated fatty acids; UFAs, unsaturated fatty acids.

**Table 3:** Characteristics of *M. speciosa* as feed.

Measured Item	Detected Value	Measured Item	Detected Value
Basic Components (DM%)		Functional Metabolites (DM%)	
Crude protein	14.2	Total phenols	6.09
WSC	5.82	Simple phenols	1.93
NDF	60.48	Hydrolyzed tannins	4.17
ADF	49.29	Condensed tannins	0.31
Moisture	4.2	Total flavonoids	4.81
Crude fat	1.4	Mineral elements (DM%)	
Crude ash	4.4	P (Total)	0.14
Acid detergent lignin	29.02		

Note: DM% indicates the percentage of dry matter content.

**Table 4:** Chemical composition of the essential oil from *M. speciosa* flowers extracted using the hydrodistillation method.

No.	RT	Compound	Molecular Formula	SI	RI ^a^	RI ^b^	Molecular Mass	Relative Percentage (%)
1	14.128	Linalool	C_10_H_18_O	96	1080	1080	154	0.52
2	16.977	*α*-Terpineol	C_10_H_18_O	87	1178	1192	154	1.49
3	18.234	2,6-Octadien-1-ol, 3,7-dimethyl-, (*Z*)-	C_10_H_18_O	96	1217	1217	154	0.27
4	18.991	2,6-Octadien-1-ol, 3,7-dimethyl-, (*E*)-	C_10_H_18_O	97	1238	1235	154	1.09
5	19.441	Nonanoic acid	C_9_H_18_O_2_	95	1244	1248	158	0.36
6	22.768	Tetradecane	C_14_H_30_	97	1426	1395 [11]	198	0.08
7	24.345	2,6,10-Trimethyltridecane	C_16_H_34_	95	1476	1462	226	0.07
8	25.273	Pentadecane	C_15_H_32_	96	1526	1496 [12]	212	0.08
9	26.262	2-Bromo dodecane	C_12_H_25_Br	80	1513	1505	248	0.43
10	26.847	Dodecanoic acid	C_12_H_24_O_2_	93	1542	1558	200	0.91
11	27.64	Hexadecane	C_16_H_34_	94	1621	1593 [11]	226	0.31
12	28.993	Bisabolol oxide B	C_15_H_26_O_2_	83	1669	1651 [13]	238	0.16
13	29.876	Heptadecane	C_17_H_36_	95	1710	1698 [14]	240	0.18
14	30.616	Cryptomerione	C_15_H_22_O	90	1725	1725	218	0.15
15	31.356	Tetradecanoic acid	C_14_H_28_O_2_	99	1747	1748	228	2.53
16	32.014	Octadecane	C_18_H_38_	95	1795	1797 [11]	254	0.15
17	32.964	Hexahydrofarnesyl acetone	C_18_H_36_O	96	1835	1843	268	1.11
18	33.339	Pentadecanoic acid	C_15_H_30_O_2_	95	1840	1833	242	0.71
19	33.535	1-Nonadecene	C_19_H_38_	93	1854	1892	266	0.37
20	34.571	Hexadecanoic acid, methyl ester	C_17_H_34_O_2_	98	1913	1915	270	0.37
21	35.308	Dibutyl phthalate	C_16_H_22_O_4_	93	1915	1922	278	0.59
22	35.858	*n*-Hexadecanoic acid	C_16_H_32_O_2_	99	1950	1946	256	21.73
23	38.506	Phytol	C_20_H_40_O	94	2086	2096	296	2.51
24	39.183	Linolenic acid	C_18_H_30_O_2_	95	2111	2116	278	0.39
25	42.778	Pentacosane	C_25_H_52_	94	2501	2501 [14]	352.407	5.86
26	45.133	Tetracosane	C_24_H_50_	98	2393	2397 [14]	338.391	19.96
27	48.638	Nonacosane	C_29_H_60_	99	2845	2895 [11]	408.47	5.16

Note: RT means retention time (min). SI means similarity index. RI means retention index; RI ^a^ was calculated from experimental data and RI ^b^ was obtained by comparison with NIST databases "https://webbook.nist.gov/chemistry/ (accessed on 20 October 2023)" or references.

## Data Availability

Data will be made available on request.

## Supplementary Materials

The following supporting information can be downloaded at: https://www.mdpi.com/article/10.3390/plants12223900/s1, Figure S1: Panoramic view and above ground portion of *Millettia speciosa* planting; Table S1: Amino acid standard weighed value and molecular weight when configuring 1 μmol/mL mixed amino acid standard stock solution; Table S2: Antibacterial activities of different extracts from *M. speciosa* (concentration of test samples 30 mg/mL); Table S3: Antibacterial activities of different extracts from *M. speciosa* (concentration of test samples 60 mg/mL); Table S4: Antibacterial activities of different extracts from *M. speciosa* (concentration of test samples 90 mg/mL); Table S5: Cytotoxic activities of different extracts from *M. speciosa*; Table S6: Antifungal activities of different extracts from *M. speciosa*; Video S1: Not applicable.
