# Supplementary material for: Nutritional Value, Volatile Components, Functional Metabolites, and Antibacterial and Cytotoxic Activities of Different Parts of Millettia speciosa Champ., a Medicinal and Edible Plant with Potential for Development
Source: Plants (Basel). 2023 Nov 19;12(22):3900. doi: 10.3390/plants12223900 (PMC10674594; doi:10.3390/plants12223900)
Supplement: Supplementary file 1 [file plants-12-03900-s001.zip › Supplementary material.pdf]

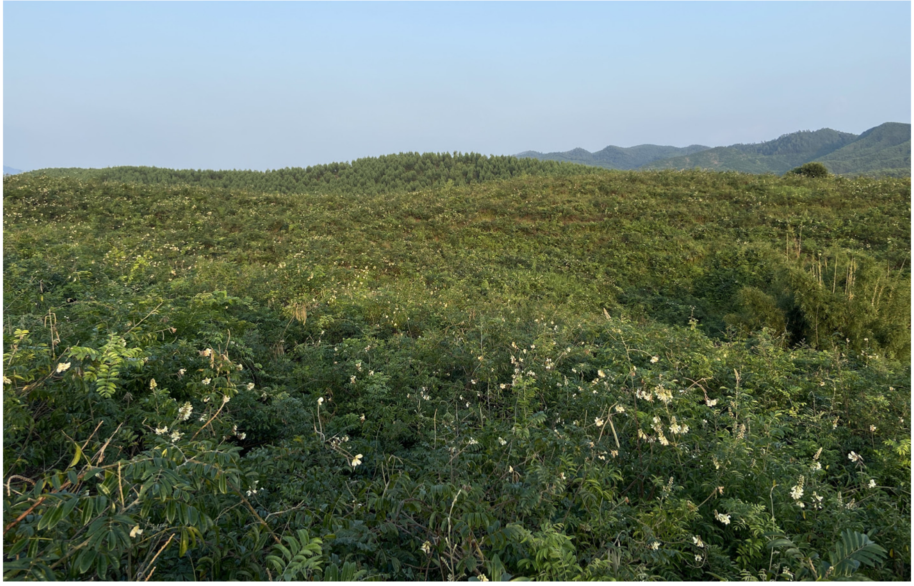

Panoramic view

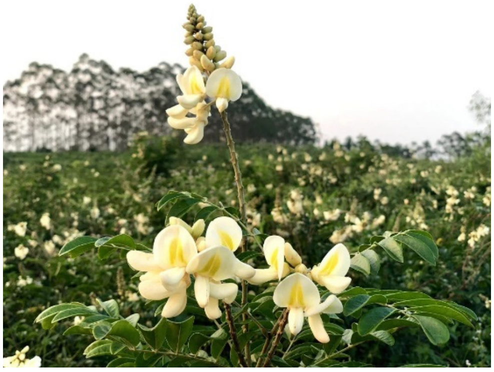

Flower

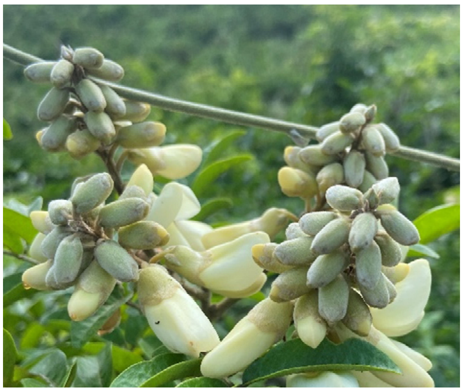

Seed and seed pod

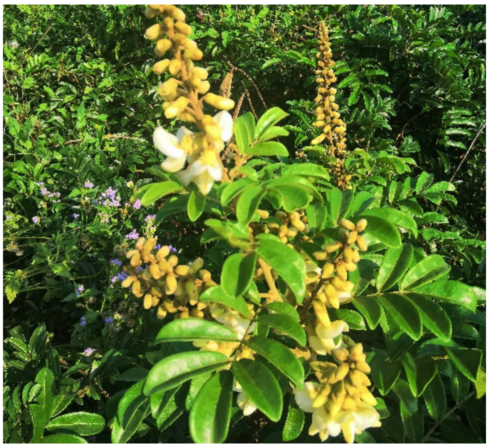

Aboveground parts

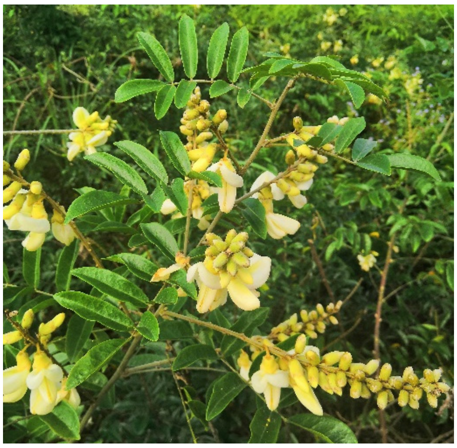

Aboveground parts

Figure S1 Panoramic view and above ground portion of *Millettia speciosa* planting

Table S1 Amino acid standard weighed value and molecular weight when configuring 1 μmol/mL mixed amino acid standard stock solution

Amino acid name	Amino acid mass (mg)	Molar mass (g/mol)
L-Aspartic acid	33	133.1
L-Threonine	30	119.1
L-Serine	26	105.1
L-Glutamic acid	37	147.1
L-Proline	29	115.1
Glycine	19	75.07
L-Alanine	22	89.06
L-Contributory	29	117.2
L-Methionine	37	149.2
L-Isoleucine	33	131.2
L-Leucine	33	131.2
L-Tyrosine	45	181.2
L-Phenylalanine	41	165.2
L-Histidine hydrochloride	52	209.7
L-Lysine hydrochloride	46	182.7
L-Arginine hydrochloride	53	210.7

Table S2 Antibacterial activities of different extracts from *M. speciosa* (concentration of test samples 30 mg/mL)

Test bacteria	Inhibitory circle diameter (mm)				
	AC	Seed pods	Flowers	Branches	Leaves
R. solanacearum	19.40 ± 0.36 d	14.43 ± 0.51 g	13.53 ± 0.40 g	9.00 ± 0.80 ij	6.00 ± 0.00 k
A. tumefaciens	26.90 ± 0.35 a	15.93 ± 0.51 f	12.30 ± 1.00 h	10.10 ± 1.13 i	6.00 ± 0.00 k
S. haemolyticus	17.57 ± 0.23 e	11.60 ± 0.26 h	12.33 ± 1.35 h	6.00 ± 0.00 k	6.00 ± 0.00 k
B. subtilis	22.83 ± 0.15 b	7.13 ± 0.12 k	11.67 ± 0.61 h	6.00 ± 0.00 kk	7.50 ± 0.40 k
X. vesicatoria	20.83 ± 0.72 c	11.80 ± 0.17 h	10.13 ± 0.12 i	8.53 ± 0.40 j	7.30 ± 0.90 k
P. lachrymans	23.57 ± 0.51 b	12.30 ± 0.50 h	14.67 ± 0.35 g	9.53 ± 2.40 ij	6.00 ± 0.00 k

Note: AC means the positive control streptomycin sulfate at 10 mg/mL.

Table S3 Antibacterial activities of different extracts from *M. speciosa* (concentration of test samples 60 mg/mL)

**Test bacteria**	**Inhibitory circle diameter (mm)**				
	AC	Seed pods	Flowers	Branches	Leaves
R. solanacearum	19.40 ± 0.36 d	18.43 ± 0.32 de	15.00 ± 0.44 f	12.43 ± 0.23 g	8.00 ± 1.11 k
A. tumefaciens	26.90 ± 0.35 a	12.83 ± 0.15 g	12.73 ± 0.75 g	10.87 ± 1.37 hi	12.03 ± 0.29 gh
S. haemolyticus	17.57 ± 0.23 e	12.73 ± 0.84 g	14.23 ± 0.06 f	9.30 ± 0.50 ij	6.00 ± 0.00 l
B. subtilis	22.83 ± 0.15 b	10.83 ± 0.96 hi	9.50 ± 0.70 ij	6.00 ± 0.00 l	10.90 ± 2.52 hi
X. vesicatoria	20.83 ± 0.72 c	15.07 ± 0.60 f	15.17 ± 0.35 f	8.97 ± 0.25 ij	11.43 ± 0.75 gh
P. lachrymans	23.57 ± 0.51 b	14.23 ± 0.06 f	19.03 ± 0.58 d	9.07 ± 0.40 ij	12.47 ± 1.82 g

Note: AC means the positive control streptomycin sulfate at 10 mg/mL.

Table S4 Antibacterial activities of different extracts from *M. speciosa* (concentration of test samples 90 mg/mL)

Test bacteria	Inhibitory circle diameter (mm)				
	AC	Seed pods	Flowers	Branches	Leaves
R. solanacearum	19.40 ± 0.36 f	18.73 ± 0.46 fg	19.67 ± 0.85 ef	15.07 ± 1.02 jk	13.73 ± 0.25 l
A. tumefaciens	26.90 ± 0.35 a	15.90 ± 0.72 ij	19.43 ± 1.00 f	10.30 ± 0.50 no	8.33 ± 0.47 p
S. haemolyticus	17.57 ± 0.23 gh	15.57 ± 1.21 ijk	15.10 ± 0.17 jk	10.10 ± 1.28 o	8.50 ± 0.30 p
B. subtilis	22.83 ± 0.15 bc	11.87 ± 0.12 m	12.10 ± 0.85 m	8.53 ± 0.64 p	12.07 ± 1.10 m
X. vesicatoria	20.83 ± 0.72 de	15.50 ± 1.20 ijk	14.40 ± 0.53 kl	11.67 ± 1.33 m	11.50 ± 0.20 mn
P. lachrymans	23.57 ± 0.51 b	16.43 ± 0.23 hi	22.00 ± 1.00 cd	11.53 ± 0.40 mn	12.27 ± 0.75 m

Note: AC means the positive control streptomycin sulfate at 10 mg/mL.

Table S5 Cytotoxic activities of different extracts from *M. speciosa*

**Extracts**	**IC_{50} value (mg/mL)**
Seed pods	0.37 ± 0.03
Flowers	0.27 ± 0.03
Leaves	0.71 ± 0.04
Branches	0.55 ± 0.02

Table S6 Antifungal activities of different extracts from *M. speciosa*

**Pathogenic fungi for testing**	**EC_{50} value (μg/mL)**			
	AC	Seed pods	Branches	Leaves
P. elaeocarpicola	0.23 ± 0.0023	139.30 ± 5.70	29.57 ± 7.20	18.28 ± 0.54
C. gloeosporioides	0.35 ± 0.035	780.23 ± 9.62	1067.91 ±42.09	568.21 ± 33.60

Note: AC means the positive control carbendazim.
